# Plumbagin protects liver against fulminant hepatic failure and chronic liver fibrosis via inhibiting inflammation and collagen production

**DOI:** 10.18632/oncotarget.12655

**Published:** 2016-10-14

**Authors:** Huafeng Wang, Huan Zhang, Yuqing Zhang, Dan Wang, Xixi Cheng, Fengrui Yang, Qi Zhang, Zhenyi Xue, Yan Li, Lijuan Zhang, Luhong Yang, Guolin Miao, Daiqing Li, Zhiyu Guan, Yurong Da, Zhi Yao, Fei Gao, Liang Qiao, Li Kong, Rongxin Zhang

**Affiliations:** ^1^ Laboratory of Immunology and Inflammation, Department of Immunology and Research Center of Basic Medical Sciences, Tianjin Medical University, Tianjin, China; ^2^ Department of Immunology, Tianjin Key Laboratory of Cellular and Molecular Immunology, Key Laboratory of Immune Microenvironment and Diseases, Ministry of Education of China, Tianjin Medical University, Tianjin, China; ^3^ School of Life Science, Shanxi Normal University, Linfen, China; ^4^ Clinical Laboratory, Tianjin Academy of Traditional Chinese Medicine Affiliated Hospital, Tianjin, China; ^5^ Department of Hepatobiliary and Pancreatic Surgery, Tianjin Nankai Hospital, Nankai Clinical School of Medicine, Tianjin Medical University, Tianjin, China; ^6^ Key Laboratory of Hormones and Development (Ministry of Health), Metabolic Diseases Hospital and Tianjin Institute of Endocrinology, Tianjin Medical University, Tianjin, China; ^7^ Department of Pathogenic Biology, Weifang Medical University, Shandong, China; ^8^ State Key Laboratory of Reproductive Biology, Institute of Zoology, Chinese Academy of Sciences, Beijing, China; ^9^ Storr Liver Unit, Westmead Millennium Institute, The Western Clinical School of the University of Sydney, Westmead, NSW, Australia; ^10^ Department of Histology and Embryology, Dalian Medical University, Dalian, China

**Keywords:** plumbagin, fulminant hepatic failure, liver fibrosis, hepatic stellate cell, inflammation

## Abstract

Plumbagin is a quinonoid constituent extracted from Plumbago genus, and it exhibits diverse pharmacological effects. This study thoroughly investigated the effects of plumbagin on thioacetamide-induced acute and chronic liver injury. Results shown that plumbagin increased survival rate, reduced liver congestion and inflammation, and decreased macrophages and neutrophils in the fulminant hepatic failure model, and remarkably diminished liver fibrosis and inflammation in the chronic liver injury model. Furthermore, plumbagin significantly suppress the HSCs/myofibroblasts activation by reduced expression of markers α-SMA and COL-1/3, and reduced macrophage in liver. In the in vitro study, plumbagin induced apoptosis and suppressed the proliferation of LX-2 cells (human HSCs). Plumbagin treatment increased AMPK phosphorylation and attenuated NF-κB, STAT3, and Akt/mTOR signals in LX-2 cells, while SMAD2 phosphorylation was not changed. Noticeably, plumbagin promoted AMPK binding to p300 which is a cofactor of SMAD complex, this may further competitively decreases the p300/SMAD complex initiated transcription of COL-1/3 and α-SMA. Additionally, plumbagin hampered inflammation related NF-κB signal in RAW 264.7 cells. In conclusion, these findings indicate that plumbagin may be a powerful drug candidate to protect the liver from acute and chronic damage by inhibiting inflammation and collagen production.

## INTRODUCTION

The liver is the largest solid organ in the body, and it plays a pivotal role in metabolism and exhibits an alexipharmic function in the body [1]. Liver damage is a major disease that seriously threatens human health worldwide, especially in Asian countries. Fulminant hepatic failure (FHF) is caused by a variety of factors, such as viral infection, drug damage and food poisoning [2]. FHF often presents with extensive hepatocyte necrosis and severe liver function abnormalities. FHF is a concern because of its acute onset, rapid progression and high fatality rate. Liver fibrosis is a common chronic liver disease that is caused by variety of pathogenic factors [3]. Liver fibrosis is not an independent disease, and it is always accompanied with chronic hepatitis.

The root of Plumbago zeylanica L has been used for centuries in traditional Indian and Chinese medicine for the treatment of various ailments. Plumbagin (5-hydroxy-2-methyl-1,4 naphthoquinone) is a quinonoid constituent that is found in the roots of medicinal herbs of the Plumbago genus [4], and it exhibits diverse pharmacological effects. Hyperlipidemic rabbits that received plumbagin exhibited a definite regression of atheroma and reduced accumulation of cholesterol and triglycerides in the liver and aorta [5]. Plumbagin induces tumor regression in 3-methyl-4-dimethyl aminoazobenzene -induced hepatoma in Wistar male rats when administered orally at a dose of 4 mg/kg body weight [6]. Plumbagin also inhibits azoxymethane-induced intestinal carcinogenesis in rats [7]. Plumbagin treatment exerted negative effects on the invasion, migration and adhesion of HepG2 cells *in vitro* by decreasing MMP-2 and u-PA expression and enhancing TIMP-2 and PAI-1 expression [8]. Previous research reported the protective effects of plumbagin on liver disease, but these studies focused more on glucose metabolism and hepatocarcinogenesis [6, 8]. Therefore, knowledge of the effects of plumbagin on FHF and liver fibrosis is very poor.

Recently, we have found that several herbal compounds derived from medicinal plants exerts potent the anti-inflammatory and anti-tumor effects [9–12], the herbal compound plumbagin exerts anti-inflammatory effects on central nervous system inflammation in experimental autoimmune encephalomyelitis [13], these suggests that plumbagin may also play anti-inflammatory role in other inflammatory diseases such as inflammatory liver injury. Thioacetamide (TAA)-induced liver injury model is widely used to study the acute-toxic liver injury and chronic liver inflammation and fibrosis. We have reported that adiponectin-derived active peptide ADP355 exerts anti-inflammatory and anti-fibrotic activities in TAA-induced liver injury [14], and mesenchymal stem cells (MSCs) secreted molecules predominantly ameliorate TAA-induced fulminant hepatic failure [15]. In this study we performed a deeper and extensive investigation of the effects of plumbagin on acute and chronic liver injury. We constructed the animal models of TAA-induced liver damage [14, 15] and examined the protective effects of plumbagin on acute and chronic liver injury in mice using inflammatory infiltration, hepatocytes protection, and fibrogenesis. We also investigated the possible molecular mechanism involved in plumbagin protective effects using LX-2 cells and RAW264.7 cells *in vitro*.

## RESULTS

### Plumbagin improved survival rate and reduced the risk of thrombosis in the FHF model

**Figure 1:**
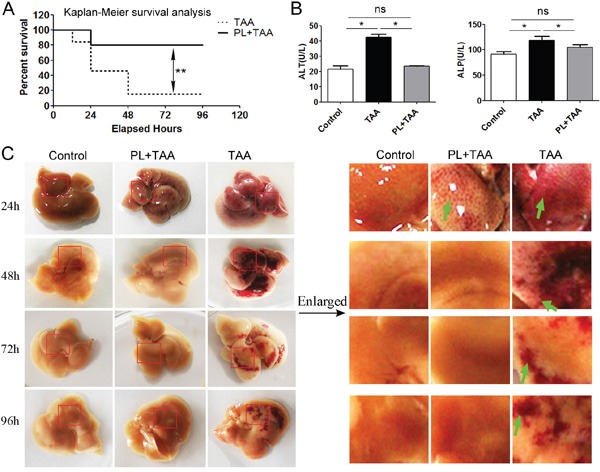
Plumbagin protected mice from fulminant hepatic failure **A.** Survival rates of PL+TAA and TAA mice. Acute liver injury was established using a single injection of 4% TAA (300 μg/g bw ip). The presence of plumbagin (2 μg/g bw ig) effectively reduced TAA-induced death. **B.** The level of ALP/ALT 96 h after intraperitoneal TAA infusions. **C.** Gross histology of livers. Acute liver injury was induced, and mice were sacrificed at the progressive daily points. Left: Gross liver histology. Right: Enlargement of liver tissues (in red circle of the left). The presence of plumbagin significantly diminished the congestion (arrows denote) due to TAA-induced hemorrhage. Representative images are shown for all panels. Mean ± SD, ns (not significant), * p<0.05, **p<0.01 were calculated using two-tailed Student's t test (T TEST).

To verify the protective effect of plumbagin on acute liver injury, plumbagin (2 μg/g bw, once daily) [16] was administered intragastrically to ICR female mice before TAA injection until the end of the experiment and 2 days later FHF model was induced using a single intraperitoneal injection of TAA (300 μg/g bw) [14, 15]. Survival rates after TAA administration in TAA mice (n=16) were 81.3% (12 h), 56.3% (24 h), and 37.5% (48 h), and remained 37.5% to 96 h later. Survival rates in PL+TAA mice (n=14) were 85.7% (24 h) and remained 85.7% to 96 h later. There was a significant difference in survival rates after TAA administration between TAA and PL+TAA mice (P= 0.013 using the log-rank test, P= 0.017 using Wilcoxon's test) (Figure 1A). ALP and ALT levels, which are markers of liver function, were higher in TAA mice 96 h after TAA administration, but these levels were lower with plumbagin treatment (Figure 1B). Livers from the TAA group were obviously congested after TAA injection, but this congestion was remarkably attenuated in PL+TAA mice (Figure 1C).

### Plumbagin inhibited the inflammatoryreaction and depressed macrophage and neutrophil in the FHF model

**Figure 2:**
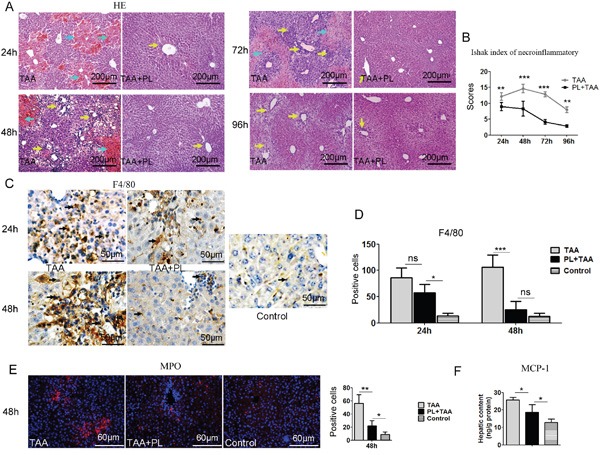
Plumbagin decreased congestion and inflammatory cell infiltration and macrophage recruitment in the FHF model **A.** Congestion (cyan arrows) and inflammatory cell infiltration (yellow arrows) was assayed using HE staining. **B.** Necroinflammatory scores were determined using the Ishak classification. **C.** Immunostaining of F4/80 (black arrows). **D.** Positive cells of F4/80 were assessed by manual method. **E.** Immunostaining of MPO. Left panel, pictures; right panel, quantification of left images. **F.** Hepatic content of MCP-1. Representative images are shown for all panels. ns (not significant), * p<0.05, **p< 0.01; ***p< 0.001. Data are expressed as means ± SD. Statistical analyses were performed using Student's t test.

Histological analyses of livers using HE staining revealed massive congestion around the central veins in TAA mice, and this congestion was attenuated in PL+TAA mice 24 h after TAA injection. Some inflammatory responses remained in TAA mice, but the primary damaged areas were repaired in PL+TAA mice 96 h after TAA administration (Figure 2A). Histology analysis by the Ishak's scoring system [14, 15] showed that necroinflammation was significantly reduced with plumbagin (Figure 2B). We next addressed whether macrophages increased in livers. The expression of F4/80 protein (macrophage marker) increased sharply in the TAA group 48 h after TAA administration. Differences between the TAA and PL+TAA groups were prominent (Figure 2C, 2D). It was demonstrated that hepatic infiltration of neutrophils (indicated as myeloperoxidase (MPO), Figure 2E) and hepatic production of monocyte chemotactic protein 1(MCP-1) (Figure 2F) were reduced with plumbagin treatment in TAA-induced fulminant liver failure model.

### Plumbagin reversed TAA-induced liver fibrosis, inflammation and liver function abnormalities in chronic liver model

**Figure 3:**
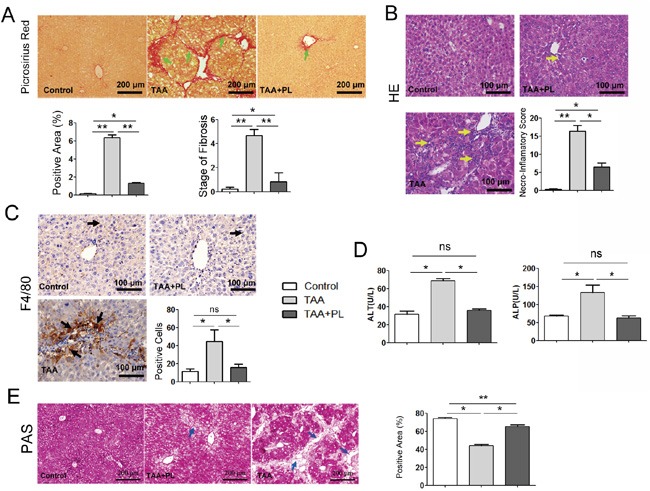
Plumbagin suppressed TAA-induced collagen deposition, inflammatory cell infiltration, and liver function abnormalities in a chronic liver damage model **A.** Fibrosis (green arrows) was determined using sirius red staining. **B.** Infiltration of immune cells (yellow arrows) was shown using HE. **C.** F4/80 protein (black arrows) was measured using immunohistochemical staining (brown, HRP-conjugated and developed using DAB). Sections were counterstained with hematoxylin (dark blue). **D.** ALP and ALT levels. **E.** Periodic acid Schiff staining for hepatocellular glycogen storage (glycogen in red). Patches of periodic acid Schiff negative, nonfunctional hepatocytes were shown by blue arrows. Histopathological evaluations were conducted using Ishak's scoring system and Image-Pro Plus 6 Windows Software. Representative images are shown for all panels. Data are expressed as means ± SD. ns (not significant), *p< 0.05; **p< 0.01. p values were calculated using two-sided Student's t tests.

To further clarify the protective effect of plumbagin on chronic liver damage, An experimental hepatic fibrosis model was established with TAA (200 μg/g bw ip, 3 times weekly) injection intraperitoneally into mice for six weeks. Plumbagin (2 μg/g/day bw ig) was administered after 6 TAA administrations until end of experiment. Liver fibrogenesis and inflammation were remarkably hampered by plumbagin treatment (Figure 3A, 3B). Liver fibrosis stage and necroinflammatory scores in the Ishak system demonstrated that the differences between TAA mice and TAA+PL mice were statistically significant (Figure 3A, 3B). The expression of the macrophage marker F4/80 was suppressed by plumbagin in TAA+PL mice (Figure 3C). The liver function abnormalities in TAA mice, such as increased serum ALP/ALT levels, were reversed by plumbagin in TAA+PL mice. Glycogen metabolism is an important function of the liver [14, 15]. A cytoplasm filled with red glycogen particles was observed using periodic acid Schiff (PAS) staining. Glycogen massively disappeared due to reduced hepatocytes in livers from TAA mice, and many of these cells reappeared in livers from TAA+PL mice (Figure 3E).

### Plumbagin therapy diminished chronic liver fibrosis by targeting HSCs/myofibroblasts

**Figure 4:**
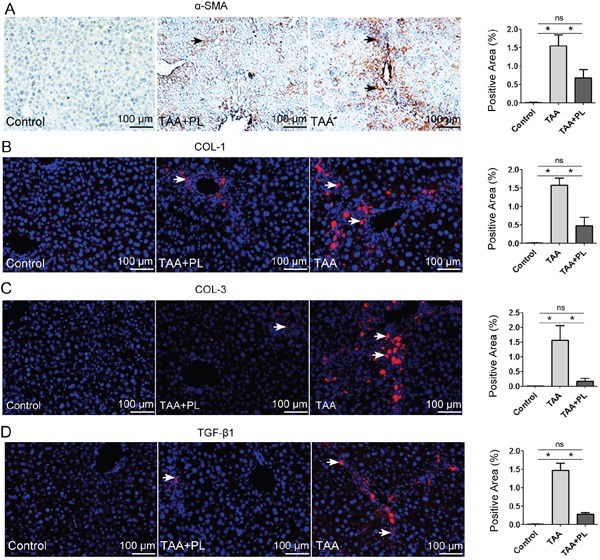
Immunofluorescence assays of HSCs/myofibroblasts **A.** α-SMA, a marker of activated HSCs/myofibroblasts; **B.** COL-1 and **C.** COL-3, markers of ECM produced by HSCs/myofibroblasts; **D.** TGF-β1. The TGF-β1 signal is involved in HSCs/myofibroblasts. HRP-(brown) or Alexa 555 (red)-conjugated secondary antibodies were used. The sections were counterstained with hematoxylin (dark blue) or DAPI (blue). Representative images are shown for all panels. The column charts given in figure are means ± SD values from three separate experiments.*p< 0.05; **p< 0.01. Statistical analyses were performed using Student's t test.

HSCs/myofibroblasts stimulate the process of liver fibrosis [14, 17]. As expected, strong positive staining for α-SMA, which is a marker of HSCs/myofibroblasts [14], was detected in TAA mice, and this staining was greatly reduced in TAA+PL mice (Figure 4A). The deposition of liver ECM indicated that the production of type 1/3 collagens was markedly suppressed in TAA+PL mice compared to TAA mice (Figure 4B, 4C). TGF-β1 signaling is required for HSC/myofibroblasts activation [14, 18]. Immunostaining revealed that plumbagin treatment decreased the expression of TGF-β1 proteins in TAA-induced chronic liver fibrosis (Figure 4D).

### Plumbagin enhanced liver regeneration and reduced liver apoptosis in the TAA-induced chronic liver fibrosis model

**Figure 5:**
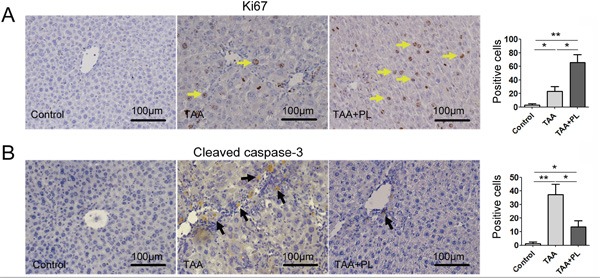
Plumbagin enhanced hepatocellular proliferation and inhibited hepatocellular apoptosis in a chronic liver fibrosis model **A.** Ki67 (yellow arrows), a marker of proliferation. **B.** Cleaved caspase-3, a marker of apoptosis (black arrows). The sections were incubated with HRP-conjugated secondary antibodies and developed using DAB (brown). The sections were counterstained with hematoxylin (dark blue). Data are expressed as means ± SD, and data are representative of 4-6 mice/group. *p< 0.05; **p< 0.01. Statistical analyses were performed using Student's t tests.

We hypothesized that the restoration of liver function in TAA+PL mice was related to liver regeneration. Liver regeneration marked by Ki67 occurred in response to damage in TAA and TAA+PL mice, but liver regeneration in TAA+PL mice was higher (Figure 5A). Liver apoptosis levels were detected using cleaved caspase-3 staining. Plumbagin therapy diminished liver apoptosis in the TAA+PL group (Figure 5B). It was suggested that PL exerts its beneficial effect on liver regeneration predominantly through enhancing the proliferation of hepatocytes.

### Plumbagin suppressed TGF-β1 signal by the activation of AMPK in human hepatic stellate cells (LX-2) *in vitro*

**Figure 6:**
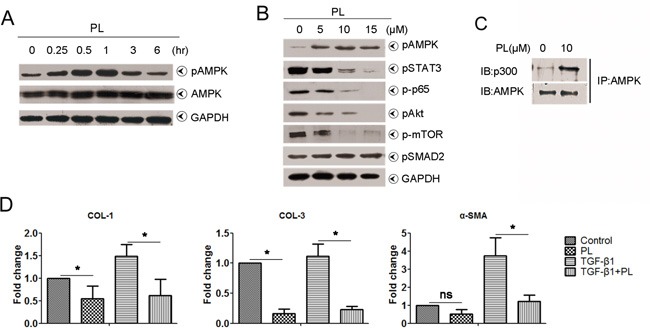
Effects of plumbagin on human hepatic stellate cells **A.** Plumbagin promoted pAMPK levels in a time-dependent manner. LX-2 cells were treated with 10 μM plumbagin for the indicated times, and pAMPK levels were analyzed. **B.** Plumbagin inhibited pSTAT3, p-p65, pAkt and p-mTOR levels in a dose-dependent manner. However, plumbagin had no effect on pSMAD2. LX-2 cells were first treated with the indicated concentrations of plumbagin for 1 h after incubation. Whole cells were harvested and analyzed using Western blots for pAMPK, pSTAT3, p-p65, pAkt, p-mTOR and pSMAD2. GAPDH was used as a loading control. **C.** Plumbagin facilitated the bind of AMPK with p300. LX-2 cells were treated with 10 μM plumbagin for 1 h, and cell lysates were immunoprecipitated using an anti-AMPK antibody, followed by immunoblotting with an anti-p300 or anti-AMPK antibody. **D.** Plumbagin decreased mRNA level of fibrosis-associated genes. LX-2 cells were treated with plumbagin (200 ng/ml) or TGF-β1 (2 ng/ml) or combination for 24 h, and then total RNA were extracted for quantitative PCR of COL-1, COL-3 and α-SMA. Representative images are shown for all panels. Data are expressed as means ± SD. *p< 0.05; **p< 0.01. p values were calculated using a two-sided Student's t test.

**Figure 7:**
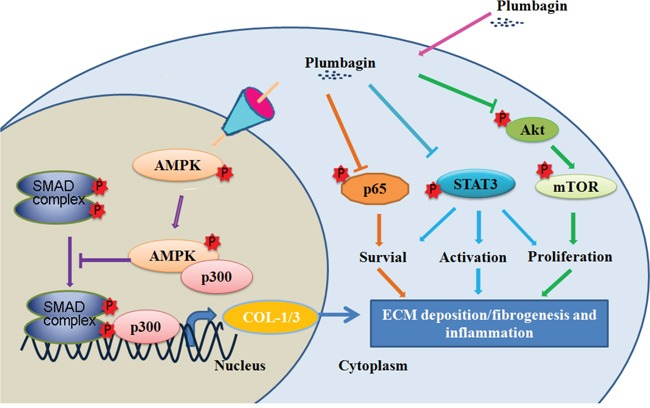
Liver protection mechanism of plumbagin Plumbagin increased AMPK phosphorylation that promoted AMPK binding to p300, which is a SMAD transcriptional cofactor. This may further competitively decreases the p300/SMAD complex initiated transcription for COL-1/3 and α-SMA. Plumbagin also attenuated NF-κB, STAT3, and Akt/mTOR signals in LX-2 cells, which were involved in pro-inflammation and survival of HSCs/myofibroblasts. In conclusion, these findings indicate that plumbagin may be a powerful drug candidate to protect the liver from acute and chronic damage by inhibiting inflammation and collagen production.

We addressed whether plumbagin suppressed HSCs *in vitro*. The inactivation of HSCs is associated AMPK [14, 19]. Plumbagin induced AMPK phosphorylation in LX-2 cells, and peak phosphorylation occurred at 1 h (Figure 6A). pSMAD2 levels were not significantly different between groups with or without plumbagin (Figure 6B). AMPK is a powerful competitor of SMAD complex for the interaction with p300 [20] and plumbagin-stimulated AMPK highly interacted with p300 (Figure 6C and Figure 7). The expression of TGF-β1 signal downstream genes (also liver fibrosis-associated genes) such as COL1, COL3 and α-SMA in LX-2 cells was greatly reduced by plumbagin (Figure 6D).

It was also observed that plumbagin sharply inhibited other signals, such as pSTAT3, NF-κB/p65, and Akt/mTOR in LX-2 cells (Figure 6B), which all are involved in inflammation and liver fibrosis [21–25]. In addition, NF-κB/p65 activation in RAW 264.7 cells was decreased by plumbagin (Supplementary Figure 1).

## DISCUSSION

Plumbago genus has been shown diverse pharmacological effects on various ailments, and its extractive, known as plumbagin [4–8]. Acute liver failure (also referred to as fulminant hepatic failure) is a rare disorder that is associated with very high mortality [2]. Chronic liver failure, featured as fibrosis, is common over the world [3]. Owing to overcapacity of liver function, people with chronic liver injury are often unconscious at beginning, and conscious until to advanced stage, featured as liver cirrhosis or developed into the hepatoma [3]. Whether liver failure is acute or chronic, orthotopic liver transplantation is the current gold standard of care, but its application is limited because of organ donor shortage, financial considerations, and the requirement for lifelong immunosuppression [2, 3]. In the present animal model of FHF, we found that plumbagin treatment protected the mice against substantial lethality, and significantly decreased serum ALT and ALP levels in the TAA-induced acute inflammatory response. Plumbagin treatment simultaneously improved histological changes in hepatic lobules, such as hemorrhaging and necrosis. In the experiments of chronic liver injury, we demonstrated that plumbagin treatment remarkably suppressed liver fibrogenesis and inflammation and promoted the restoration of liver function indicated with serum ALP/ALT levels and glycogen metabolism, which related with liver regeneration and apoptosis [14, 15]. Stimulation of hepatocytes proliferation is tightly correlated with the restoration of liver structure and function. Our results also showed that expression of Ki67 (marked as liver regeneration) increased while cleaved caspase-3 (marked as liver apoptosis) decreased in livers with plumbagin treatment. Taken together, it was suggested that plumbagin can exert pharmacological effects on acute and chronic liver injury. However, in the present study, action of plumbagin was prophylactic in FHF model and detail therapeutic effects will be needed to examine in the future.

The massive infiltration of inflammatory cells is generally accompanied with the recruitment of macrophages in acute and chronic liver damage [15, 26]. Macrophages play a crucial role in the regulation of liver inflammation [26]. The number of macrophages rises significantly in damaged livers, and these cells primarily distribute over regions of injury and fibrosis [14, 15, 27, 28]. In addition, neutrophils are also correlated with initiation of liver injury and inflammatory responses in experimental model of acute liver failure [29, 30]. Our study demonstrated a significant increase in macrophage recruitment and other inflammatory cell infiltration such as neutrophil, and related inflammatory cytokine MCP-1, as a result of liver damage, and plumbagin treatment strikingly decreased these factors in acute and chronic liver injury models. The pro-inflammatory function of hepatic macrophages was associated with the NF-κB signaling pathway in an alcohol-induced inflammation model [31]. Plumbagin suppressed the NF-κB signaling pathway in RAW264.7 cells *in vitro* in our study, which is consistent with previous studies [32, 33]. These results support the conclusion that plumbagin inhibits macrophage recruitment and suppresses NF-κB activation in macrophages.

Liver fibrosis results from the deposition of extracellular matrix proteins that are primarily produced by α-SMA-positive HSCs/myofibroblasts [14, 17]. The present study demonstrated that plumbagin treatment suppressed HSCs/myofibroblasts activation (measured as α-SMA) in livers *in vivo* and the induction of LX-2 cell apoptosis *in vitro*. Plumbagin therapy remarkably diminished TAA-induced fibrogenesis, which was verified using picrosirius red staining, the Ishak fibrosis score and immunostaining for COL-1/3 in liver sections from mice in the present experimental model. TGF-β1 signaling is tightly associated with the fibrotic response. In the present study, we found that plumbagin treatment reduced expression of TGF-β1 in livers.

AMPK activation is relevant for the development of hepatic fibrosis due to its inactivation of HSCs. Activation of AMPK suppresses the activity of HSCs by inhibiting platelet-derived growth factor (PDGF)-induced mitogenesis and migration, in addition to downregulating monocyte chemoattractant protein-1 (MCP-1) protein secretion [19]. Activation of AMPK reduces HSC proliferation and sensitizes activated HSCs to apoptosis by modulating suppressors of cytokine signaling (SOCS-3) expression [34]. In our recent study, we found that activation of AMPK can dampen the activation and proliferation of hepatic stellate cells induced by TGF-β1 [14, 15]. AMPK activation can interrupt SMAD complex association with p300 [18], which, as a SMAD transcriptional cofactor, is required by the induction of fibrosis gene expression by SMAD complex [20, 35]. In the present study, we showed the highly affinity of AMPK to p300 in the presence of plumbagin *in vitro*. Moreover, plumbagin decreased mRNA level of fibrosis-associated genes, such as COL1, COL3 and α-SMA, which all were downstream gene of TGF-β1 pathway.

NF-κB is a previously confirmed HSC survival factor [36]. Macrophages also contribute to liver fibrosis via the promotion of NF-κB activation in HSCs [21]. The Akt/mTOR pathway correlates HSC survival and proliferation [22–24]. Activated Akt is a key survival factor that directly phosphorylates mTOR, which ultimately stimulates HSC proliferation [25]. STAT3 actively promotes HSC activation [37, 38]. Plumbagin treatment inhibited all of the aforementioned signals that are associated with HSCs activation in LX-2 cells *in vitro*. These results support that plumbagin inhibited liver fibrogenesis by targeting HSCs.

In summary, the present study demonstrated that plumbagin can exert protective effects on liver injury. As summarized in Figure 7, plumbagin increased survival rate, reduced liver congestion and inflammation, and blocked the recruitment of macrophages in the FHF model. In addition, plumbagin treatment remarkably diminished liver fibrosis and inflammation in the chronic liver injury model. Furthermore, plumbagin significantly suppress the HSC/myofibroblasts activation by reduced expression of markers α-SMA and COL-1/3, and reduced F4/80 positive macrophage in liver. Plumbagin increased AMPK phosphorylation that promoted AMPK binding to p300, which is a SMAD transcriptional cofactor. This decreases the p300/SMAD complex initiated transcription for COL-1/3 and α-SMA. Moreover, plumbagin attenuated NF-κB, STAT3, and Akt/mTOR signals in LX-2 cells, which were involved in pro-inflammation and survival of HSCs. In addition, NF-κB signal also hampered with plumbagin. It is expected that plumbagin may be a powerful candidate to protect the liver from acute and chronic damage by inhibiting inflammation and collagen production.

## MATERIALS AND METHODS

### Reagents and animals

TAA, sirius red, and a 10% formalin solution were purchased from Sigma Aldrich (St. Louis, MO, USA). Plumbagin (J&K, Beijing, China) was prepared in dimethyl sulfoxide (DMSO) and diluted with PBS. ICR female mice were purchased from the Academy of Military Medical Science (Beijing, China) and weighed 20-22 g at the time of experiments. All mice were acclimatized for 1 week prior to the beginning of all experiments. Mice were housed in an animal room with a 12-hour light/dark cycle. All experimental protocols were approved by Tianjin Medical University Animal Ethics Committee and all methods were carried out in accordance with the approved guidelines.

### Experimental model

FHF model (diagrammed in Supplementary Figure 2A) was induced using a single intraperitoneal injection of TAA (300 μg/g bw) [14, 15]. Plumbagin (2 μg/g bw) [16] was administered intragastrically to PL+TAA mice 2 days before TAA injection until the end of the experiment.

Chronic liver fibrosis model [14, 15] (diagrammed in Supplementary Figure 2B). TAA (100 μg/g bw ip, 3 times weekly) was injected intraperitoneally into TAA+PL and TAA mice for the first two weeks, and the dose of TAA was increased to 200 μg/g until the sixth week. Plumbagin (2 μg/g/day bw ig) was administered after 6 TAA administrations. Mice were randomized into 3 groups: (i) Control group; (ii) TAA+PL mice, which were treated with TAA and plumbagin; and (iii) TAA mice, which were treated with TAA only.

All mice were sacrificed at specific times after TAA administration.

### ALT and ALP

Serum samples were collected using orbital venous plexus bleeding in anesthetized mice. Alanine transaminase (ALT) and alkaline phosphatase (ALP) activities were determined using an EnzyChromTM Alanine Transaminase Assay Kit (BioAssay Systems) and a QuantiChromTM Alkaline Phosphatase Assay Kit (BioAssay Systems) [14, 15], respectively.

### Histology

Livers were perfused with PBS and removed. Images were obtained, and livers were weighed and cut into pieces. Liver specimens were fixed in a neutral-buffered formalin solution, embedded in paraffin, and cut into 7 μm sections.

Liver slices were stained with hematoxylin-eosin (HE) to identify inflammation and necrosis, picrosirius red for fibrosis identification and Necroinflammation and fibrosis were scored using an Ishak system [14, 15, 39] (Details are described in Supplementary Table 1, 2). Liver slices were stained with periodic acid–Schiff (PAS) staining for analyses of glycogen metabolism. Positive staining was quantified using Image-Pro Plus 6 Windows Software (Media Cybernetics, USA).

### Immunostaining

Tissue sections were incubated with the following primary antibodies: rabbit anti-α-SMA (1:200; Abcam), anti-F4/80 (AbD Serotec, Oxford, UK), anti-myeloperoxidase antibody (1:200; Abcam), rabbit anti-collagen 1 (1:200; Abcam), anti-collagen 3 (1:1,000; Abcam), rabbit anti-TGFβ1 (1:100; Sigma-Aldrich), anti-Ki67 (Abcam, ab66155), and anti-cleaved caspase-3 (Asp 175) (Cell Signaling Technology, Inc., CAT#: 9664). Sections for immunohistochemistry were incubated with horseradish peroxidase (HRP)-conjugated secondary antibodies (1:1000; Jackson, 11-GAR007, distributed by MμltiSciences Biotech Co., Ltd.) and developed using 3,3′-diaminobenzidine (DAB). Alexa 555-conjugated secondary antibodies (1:200; Molecular Probes) were used for immunofluorescence. Sections were counterstained with hematoxylin or DAPI (eBioscience, 00-4959). Positive staining was quantified by manual method or Image-Pro Plus 6 Windows Software (Media Cybernetics, USA).

### Hepatic MCP-1 assay

Liver tissues were homogenized in RIPA (Applygen Technologies Inc, Beijing, China) by Bullet Blender Homogenizer (USA). Hepatic MCP-1 content was determined with Mouse monocyte chemotactic protein 1 ELISA Kit (Suobio, China). The procedures are according to manufacturers' protocols.

### Western blotting

Total protein from LX-2 were extracted using RIPA Lysis Buffer (Beijing Biomed) and the protease inhibitor PMSF, and the nuclear proteins were extracted using the Nucleoprotein Extraction Kit (Sangon Biotech, Shanghai). Cell lysates were separated using SDS–PAGE and transferred to Immobilon-P membranes (Millipore). Membranes were incubated with primary antibodies for AMPK (Cell Signaling Technology), pAMPK (Cell Signaling Technology), pSTAT3 (Cell Signaling Technology), pAkt (Cell Signaling Technology), p-mTOR (Cell Signaling Technology), p-SMAD2 (Sigma-Aldrich), and GAPDH (Santa Cruz Biotechnology) followed by HRP-conjugated secondary antibodies (1:2,000; Sungene Biotech Co., Ltd) that were specific to the species of the primary antibodies.

### Immunoprecipitation

The primary antibodies were AMPK and p300 (Cell Signaling Technology), and HRP-conjugated secondary antibodies (1:2,000; Sungene Biotech Co., Ltd.) that were specific to the species of the primary antibodies were used. Immunoprecipitation was performed in accordance with the protocol of Dynabeads® Protein G (Life Technologies AS, Norway).

### Gene expression

LX-2 cells were treated with plumbagin (200 ng/ml) or TGF-β1 (2 ng/ml) or combination for 24 h, and then total RNA was extracted by using Trizol reagent according to the manufacturer's instructions (Invitrogen, Carlsbad, USA). The RNA was converted into cDNA with random hexamers and M-MLV reverse transcriptase (Invitrogen, Oregon, USA). PCR was performed for COL-1/3, α-SMA and GAPDH detection. Primers were from BGI, China (Supplementary Table 3).

### Statistical analysis

Data are expressed as means ± SD (standard deviation). Statistical comparisons between experimental groups were performed using Student's t-test.

## SUPPLEMENTARY DATA


