# Supplementary material for: Plumbagin protects liver against fulminant hepatic failure and chronic liver fibrosis via inhibiting inflammation and collagen production
Source: Oncotarget. 2016 Oct 14;7(50):82864–75. doi: 10.18632/oncotarget.12655 (PMC5347738; doi:10.18632/oncotarget.12655)
Supplement: Supplementary file 1 [file oncotarget-07-82864-s001.pdf]

Plumbagin protects liver against fulminant hepatic failure and chronic liver fibrosis via inhibiting inflammation and collagen production

SUPPLEMENTARY DATA

Western blotting

Total protein from RAW264.7 cells were extracted using RIPA Lysis Buffer (Beijing Biomed) and the protease inhibitor PMSF. Cell lysates were separated using SDS–PAGE and transferred to Immobilon-P membranes (Millipore). Membranes were incubated with primary antibodies for p-p65 (Cell Signaling Technology) and GAPDH (Santa Cruz Biotechnology) followed by HRP-conjugated secondary antibodies (1:2,000; Sungene Biotech Co., Ltd) that were specific to the species of the primary antibodies.

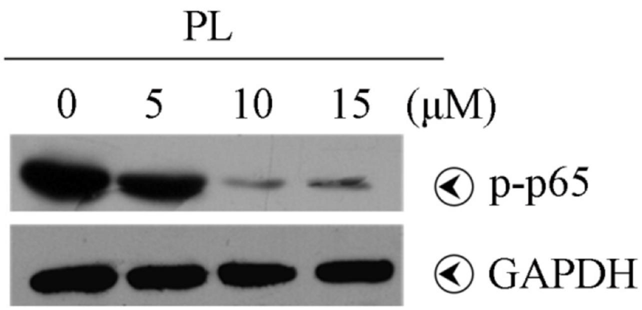

Supplementary Figure S1: The effects of plumbagin on NF-κB signal. The NF-κB signal (marked as p-p65) in RAW264.7 cells was suppressed by plumbagin treatment for 1 h at the indicated concentrations. Representative images are shown.

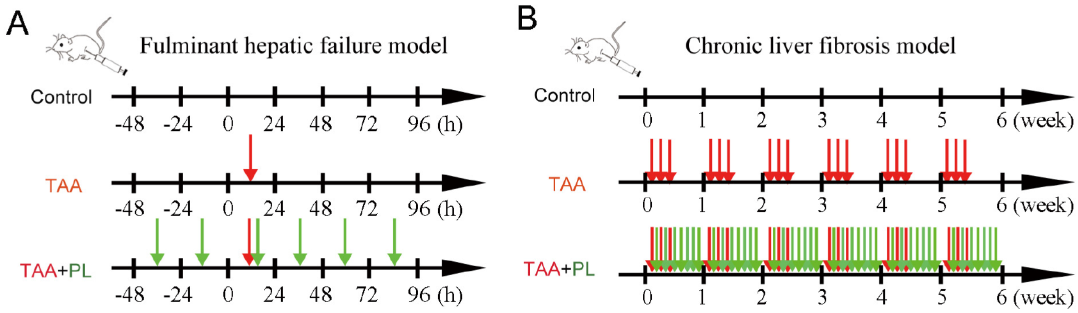

Supplementary Figure S2: Diagram of the experimental model. A. The FHF model. **B.** The chronic liver fibrosis model. TAA, red arrow; PL (plumbagin), green arrow.

Supplementary Table S1: Necroinflammatory scores system (Ishak, et al, 1995)

A	Score	Pathology
Periportal or periseptal interface hepatitis (piecemeal necrosis)	0	Absent
	1	Mild (focal, few portal areas)
	2	Mild/moderate (focal, most portal areas)
	3	Moderate around less than 50% of tracts or septa/P-P bridging necrosis)
	4	Severe (continuous around more than 50% of tracts or septa/P-P bridging necrosis)
B	Score	Pathology
Confluent necrosis	0	Absent
	1	Focal confluent necrosis
	2	Zone 3 necrosis in some areas
	3	Zone 3 necrosis in most areas
	4	Zone 3 necrosis, plus occasional portal-central (P-C) bridging
	5	Zone 3 necrosis, plus multiple P-C bridging
	6	Panacinar or multiacinar necrosis
C	Score	Pathology
Focal (spotty) lytic necrosis, apoptosis and focal inflammation	0	Absent
	1	One focus or less per 10× objective (ob)
	2	Two to four foci per 10× ob
	3	Five to ten foci per 10× ob
	4	More than ten foci per 10× ob
D	Score	Pathology
Portal inflammation	0	None
	1	Mild, some or all portal areas
	2	Moderate, some or all portal areas
	3	Moderate/marked, all portal areas
	4	Marked, all portal areas

Necroinflammatory scores=A+B+C+D.

Supplementary Table S2: Ishak index for staging of liver fibrosis

Score	pathology
0	No fibrosis
1	Some portal tract fibrotic ± short fibrous septa
2	Most portal tract fibrotic ± short fibrous septa
3	Most portal tract fibrotic with Occasional P-P bridging
4	Portal tract fibrotic with marked P-P and P-C bridging
5	Marked P-P and/or P-C bridging with occasional nodules (incomplete cirrhosis)
6	Cirrhosis (probable or definite)

Supplementary Table S3: Primers for PCR with LX-2 cells

Genes		Sequences
Homo sapiens GAPDH	Forward Primer	GGAGCGAGATCCCTCCAAAAT
	Reverse Primer	GGCTGTTGTCATACTTCTCATGG
COL-1	Forward Primer	GAGGGCCAAGACGAAGACATC
	Reverse Primer	CAGATCACGTCATCGCACAAC
COL-3	Forward Primer	TTGAAGGAGGATGTTCCCATCT
	Reverse Primer	ACAGACACATATTTGGCATGGTT
α-SMA	Forward Primer	CTATGAGGGCTATGCCTTGCC
	Reverse Primer	GCTCAGCAGTAGTAACGAAGGA
